# Supplementary material for: The Construction and Exploration of a Comprehensive MicroRNA Centered Regulatory Network in Foxtail Millet (Setaria italica L.)
Source: Front Plant Sci. 2022 May 6;13:848474. doi: 10.3389/fpls.2022.848474 (PMC9121102; doi:10.3389/fpls.2022.848474)

T=Seita.8G051500.1_Q=Sit−miRNovel6_S=1353

category=2_p=0.811813494151863

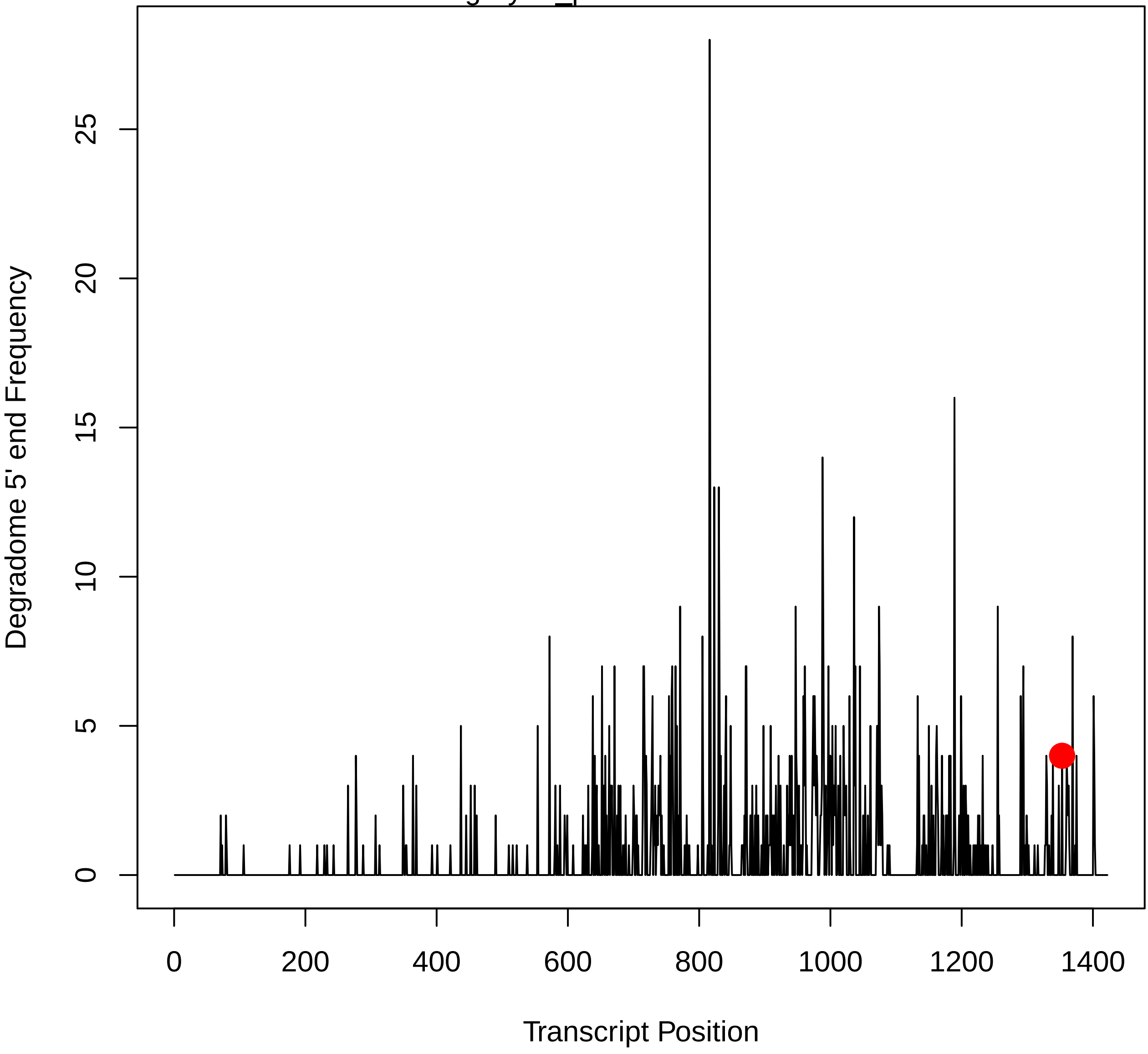

Supplement: Supplementary file 3 [file Data_Sheet_3.zip › Sit-miRNovel6_Seita.8G051500.1_1353_TPlot.pdf]
